# Intervention with fructooligosaccharides, *Saccharomyces boulardii*, and their combination in a colitis mouse model

**DOI:** 10.3389/fmicb.2024.1356365

**Published:** 2024-05-21

**Authors:** Yan Wu, Hao Fu, Xu Xu, Hui Jin, Qing-jun Kao, Wei-lin Teng, Bing Wang, Gang Zhao, Xiong-e Pi

**Affiliations:** ^1^Hangzhou Center for Disease Control and Prevention, Hangzhou, Zhejiang, China; ^2^Institute of Plant Protection and Microbiology, Zhejiang Academy of Agricultural Sciences, Hangzhou, China; ^3^School of Food Science and Biotechnology, Zhejiang Gongshang University, Hangzhou, Zhejiang, China; ^4^Institute of Rural Development, Zhejiang Academy of Agricultural Sciences, Hangzhou, China

**Keywords:** fructooligosaccharides, *Saccharomyces boulardii*, gut microbiota, colitis, inflammatory bowel disease

## Abstract

**Objective:**

To examine the effects of an intervention with fructooligosaccharides (FOS), *Saccharomyces boulardii*, and their combination in a mouse model of colitis and to explore the mechanisms underlying these effects.

**Methods:**

The effects of FOS, *S. boulardii*, and their combination were evaluated in a DSS-induced mouse model of colitis. To this end, parameters such as body weight, the disease activity index (DAI), and colon length were examined in model mice. Subsequently, ELISA was employed to detect the serum levels of proinflammatory cytokines. Histopathological analysis was performed to estimate the progression of inflammation in the colon. Gas chromatography was used to determine the content of short-chain fatty acids (SCFAs) in the feces of model mice. Finally, 16S rRNA sequencing technology was used to analyze the gut microbiota composition.

**Results:**

FOS was slight effective in treating colitis and colitis-induced intestinal dysbiosis in mice. Meanwhile, *S. boulardii* could significantly reduced the DAI, inhibited the production of IL-1β, and prevented colon shortening. Nevertheless, *S. boulardii* treatment alone failed to effectively regulate the gut microbiota. In contrast, the combined administration of FOS/*S. boulardii* resulted in better anti-inflammatory effects and enabled microbiota regulation. The FOS/*S. boulardii* combination (10^9^ CFU/ml and 10^7^ CFU/ml) significantly reduced the DAI, inhibited colitis, lowered IL-1β and TNF-α production, and significantly improved the levels of butyric acid and isobutyric acid. However, FOS/*S. boulardii* 10^9^ CFU/ml exerted stronger anti-inflammatory effects, inhibited IL-6 production and attenuated colon shortening. Meanwhile, FOS/*S. boulardii* 10^7^ CFU/ml improved microbial regulation and alleviated the colitis-induced decrease in microbial diversity. The combination of FOS and *S. boulardii* significantly increased the abundance of *Parabacteroides* and decreased the abundance of *Escherichia–Shigella*. Additionally, it promoted the production of acetic acid and propionic acid.

**Conclusion:**

Compared with single administration, the combination can significantly increase the abundance of beneficial bacteria such as *lactobacilli* and *Bifidobacteria* and effectively regulate the gut microbiota composition. These results provide a scientific rationale for the prevention and treatment of colitis using a FOS/*S. boulardii* combination. They also offer a theoretical basis for the development of nutraceutical preparations containing FOS and *S. boulardii*.

## 1 Introduction

Inflammatory bowel disease (IBD) is a chronic inflammatory condition affecting the intestinal tract, and it includes ulcerative colitis and Crohn’s disease (Wu et al., 2021). The clinical symptoms of IBD include celialgia, diarrhea, hematochezia, weight loss, and oxidative stress and patients with IBD are at an increased risk of colon cancer (Wang et al., 2019). The prevalence of IBD has been increasing year after year, making this disease an important global public health issue (Shi et al., 2020; Dong et al., 2022).

Several factors contribute to the pathogenesis of IBD including genetic susceptibility, intestinal barrier injury, environmental risk factors, and an imbalance of symbiotic flora (Liu et al., 2022; Yang et al., 2022). The three main goals of IBD management are to induce and maintain remission; prevent disease-related complications; and improve quality of life and minimize adverse events. The treatment strategies for IBD are based on the location and severity of inflammation, and account for the expected progression of the disease (Taleban et al., 2015). At present, colitis is mainly treated using clinical agents such as aminosalicylates, corticosteroids, immunosuppressants, and monoclonal antibodies. Of these, aminosalicylates are used to treat mild-to-moderate IBD. However, they cause adverse effects such as headache, nausea, and fatigue in 10 to 45% of treated patients. Additionally, they can induce allergic reactions such as rash, fever, Stevens-Johnson syndrome, hepatitis, pneumonitis, hemolytic anemia, and bone marrow suppression (Pithadia and Jain, 2011). Corticosteroids are effective at establishing but not maintaining remission in cases of moderate-to-severe IBD (Kornbluth et al., 2010). However, long-term corticosteroid use makes patients vulnerable to systemic side effects such as an increased risk of osteoporosis, opportunistic infections, and death (Toruner et al., 2008). Immunosuppressant drugs can serve as invaluable adjunct therapeutic agents for patients with intractable IBD or complex, inoperable perianal disease. Although immunosuppressant treatment causes some adverse effects, it is safer and more tolerable than long-term steroid hormone therapy. Notably, the adverse effects of immunosuppressants are rare and include leukopenia and hypersensitive interstitial pneumonitis, although long-term treatment can cause hepatic fibrosis (Pithadia and Jain, 2011). Monoclonal antibodies can also be an option in moderate-to-severe IBD, especially in steroid-dependent or steroid-resistant patients. Cottone et al. reported a 12% risk of serious infections including pneumonia and sepsis, in older patients under treatment with monoclonal antibodies. Overall, 3% of the patients in their cohort died due to septic shock (Cottone et al., 2011). In other studies, monoclonal antibodies have also been reported to cause dermatological problems, infusion reactions, and neurological sequelae. Hence, the current drugs used to treat colitis have several disadvantages such as serious adverse effects, and are not suitable for long-term use (Jeong et al., 2019; Qu Y. et al., 2021).

Fructooligosaccharides (FOS) are a typical prebiotics that can increase the diversity of the human intestinal microbiota, selectively promoting the growth of beneficial bacteria in the human gut, improving the intestinal environment and intestinal function, and decreasing the level of proinflammatory cytokines (Genda et al., 2017; Mao et al., 2018; Tandon et al., 2019). Relevant studies have shown that pretreatment with a mixture of FOS and galactooligosaccharides (GOS) can alleviate the symptoms of dextran sulfate sodium (DSS)-induced colitis in mice (Liu et al., 2021).

*Saccharomyces boulardii* is a subspecies of *Saccharomyces* yeast, and it is the only probiotic species of yeast discovered to date (Pais et al., 2020). Research on *S. boulardii* has gradually expanded in recent years, and many studies have shown that this yeast shows good efficacy in the treatment of various gastrointestinal diseases (Gao et al., 2023; Salazar-Parra et al., 2023).

Given the complexity of colitis and its etiology, treatment with FOS and *S. boulardii* alone has shown limited anti-colitis efficacy (Sivananthan and Petersen, 2018; Simoes et al., 2022). Moreover, there have been few studies on the combination of FOS and *S. boulardii* for colitis management. Hypothetically, the combination of FOS and *S. boulardii* could simultaneously provide a prebiotic and probiotic agent, synergistically enhancing the efficacy of colitis treatment (Wasilewski et al., 2015). Exploring the effects of this synbiotic combination in mouse models of colitis could provide a theoretical basis for its clinical development and application and create new therapeutic avenues for colitis management.

In this study, mice were pretreated with FOS, different concentrations of *S. boulardii*, and a combination of these agents. They were then treated with DSS to induce colitis. Parameters such as the disease activity index (DAI), the levels of SCFAs and proinflammatory cytokines, inflammation in the colon, and the composition of the gut microbiota were evaluated in the model mice after treatment. Accordingly, the preventive and therapeutic effects of FOS, *S. boulardii*, and their combination on colitis were evaluated and their regulatory effects on the mouse gut microbiota were delineated.

## 2 Experimental materials and instruments

### 2.1 Animals, microbes, reagents, and materials

The following animals, microbes, reagents, and materials were used in this study: male C57BL/6 J mice (Jiangsu GemPharmatech Co., Ltd. license NO: SYXK (Zhejiang) 2020–0022), *S. boulardii* (Angel Nutritech Co., Ltd. CAT.NO: 22011001), FOS (Baolingbao Biological Co., Ltd. CAT.NO: 22021012), DSS (MP Biomedicals Co., Ltd. CAT.NO: S7708), fecal occult blood qualitative detection kit (Shanghai Yuanye Bio-Technology Co., Ltd. CAT.NO: M03HR177092), and mouse tumor necrosis factor α (TNF-α) kit, interleukin-6 (IL-6) kit, and interleukin-1β (IL-1β) kit (Shanghai Enzyme-linked Biotechnology Co., Ltd. CAT.NO: YJ002095, YJ063159, YJ301814).

### 2.2 Instruments

The following equipment was used in this study: GC-2010 plus Gas Chromatographic instrument (Shimadzu Co., Ltd., Japan), DB-FFAP gas chromatographic column (Agilent Technologies, Inc., USA), JB-P5 embedding machine, JJ-12 J dehydrator, JB-L5 freezing table (Wuhan Junjie Electronics Co., Ltd.), KD-P water bath-slide drier (Jinhua Kedi Instrument Equipment Co., LTD.), RM2016 microtome (Leica Instrument Co., LTD.), DP260s dyeing machine (Shenzhen Dakewe Medical Technology Co., LTD.), ECLIPSE E100 microscope (NIKON Company, Japan), and Spectramax M2 Full Wavelength enzyme-labeling Apparatus (Molecular Devices, USA).

### 2.3 Methods

#### 2.3.1 Mice and housing conditions

Forty-two healthy male SPF C57BL/6 J mice (8 weeks old; average body weight, 25.25 ± 0.7 g) were housed in cages at a room temperature of 21–25°C and relative humidity of 40–70%. The lights were artificially controlled to maintain a 12-h light/dark cycle, and the corncob bedding was changed twice a week.

#### 2.3.2 Experimental protocol

**Figure 1:**
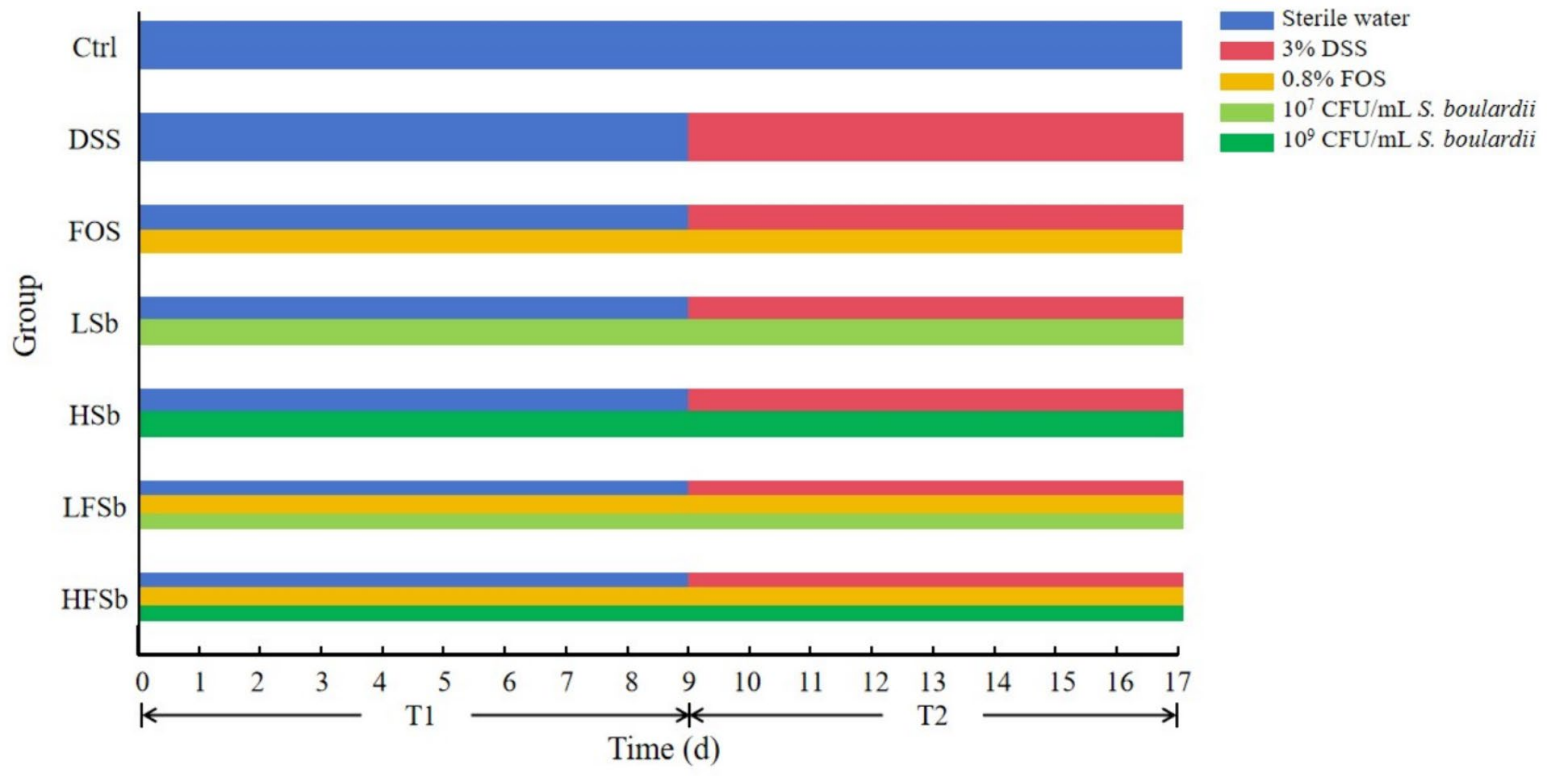
Schematic diagram of the experimental design.

The mice were randomly divided into seven groups of six mice each. These included the control group, DSS group, HSb group (High-*S. boulardii*), LSb group (Low-*S. boulardii*), FOS group, HFSb group (High-*S. boulardii* + FOS). and LFSb group (Low-*S. boulardii* + FOS). The experiment was carried out over a total duration of 17 days, including stage T1 (days 1–9) and stage T2 (days 10–17). On day 17, the mice were sacrificed, and samples (Stool, blood, and colon) were collected. Each treatment agent was administered to the mice via oral gavage (0.15 ml). The Ctrl group received the intragastric administration of sterile water, whereas the DSS group received sterile water at the T1 stage and 3% DSS at the T2 stage. Throughout the experimental period, the HSb group and the LSb group received 10^9^ CFU/ml and 10^7^ CFU/ml *S. boulardii* suspensions, respectively. Meanwhile, the FOS group was given a 0.8% FOS solution. The HFSb and LFSb groups received 0.8% FOS + 10^9^ CFU/ml *S. boulardii* suspension and 0.8% FOS + 10^7^ CFU/ml *S. boulardii* suspension, respectively. In addition, the Ctrl group was allowed to drink water freely throughout the experiment. Meanwhile, the other six groups drank water freely during the T1 stage, and the drinking water was changed to a 3% DSS solution (0.03 g/ml) during the T2 stage to induce colitis (Figure 1).

The DAI was recorded every day during the T2 stage. At the end of the experiment, stool samples were obtained to detect their short-chain fatty acid (SFCA) content. Moreover, blood samples were collected to detect serum proinflammatory cytokine levels, and colon contents were obtained for bacterial community analysis via 16S rRNA gene sequencing. Colon tissue samples were collected and stained for histological analysis.

#### 2.3.3 Preparation of *Saccharomyces boulardii* suspensions

*Saccharomyces boulardii* was cultured in yeast liquid medium at 32°C and 160 rpm for 36 h. Subsequently, the culture medium was centrifuged at 4°C and 3,000 rpm for 5 min. The supernatant was discarded, and the pellet was suspended in sterile PBS. The procedure was repeated twice to remove any residual medium. Finally, the pellet was resuspended in sterile PBS, and the number of *S. boulardii* cells was measured using a hemocytometer. Finally, *S. boulardii* suspensions with concentrations of 10^9^ CFU/mL and 10^7^ CFU/mL were prepared.

#### 2.3.4 Detection of proinflammatory cytokines in the serum

To detect the levels of TNF-α, IL-6, and IL-1β in the serum, standard, blank, and sample wells were set up on ELISA plates. Then, 50 μl of a standard solution was added to the standard well, 100 μl of the sample diluent was added to the blank well, and 40 μl of the sample diluent and 10 μl of serum were added to the sample well. The contents of each well were mixed, and subsequently, 100 μl of conjugate reagent was added to all wells except the blank wells. After incubation at 37°C for 60 min, the plates were washed five times. Then, 50 μl of color developer A and B were added successively to each well, and the color was developed at 37°C for 15 min, away from light. At the end of the color development phase, 50 μl of stop buffer was added to each well, and the absorbance of each well was measured at 400 nm. A standard curve was drawn, and the serum concentrations of the three proinflammatory cytokines were calculated.

#### 2.3.5 Preparation, staining and histopathological analysis of colon sections

The colon tissues were fixed in formaldehyde and subsequently embedded in paraffin. Paraffin-embedded specimens were cut into 4-μm-thick sections and stained with hematoxylin and eosin (H&E). The presence and extent of neutrophil infiltration, cryptitis, crypt abscesses, epithelial erosion, and ulceration in the colon sections were evaluated based on the modified Riley score. The modified Riley score classifies histological activity as follows: 0 for neutrophils present in the epithelium but no crypt involvement; 1 for neutrophils present in the epithelium and <25% crypt involvement; 2 for neutrophils present in the epithelium and ≥25% to ≤75% crypt involvement; 3 for neutrophils present in the epithelium and > 75% crypt involvement; 4 for neutrophils present in the lamina propria with a mild but unequivocal increase; 5 for neutrophils present in the lamina propria with a moderate increase; 6 for neutrophils present in the lamina propria with a marked increase; and 7 for erosion or ulceration (Ates et al., 2022).

#### 2.3.6 Detection of SCFAs in fecal samples

Mouse feces were mixed with sterile water (1:10) and centrifuged for 5 min (4°C, 10000 rpm). Subsequently, the SCFA content in the supernatant was determined using gas chromatography. The column temperature was maintained at 80°C for 1 min and then increased to 190°C at a rate of 10°C/min. It was further increased to 240°C at 40°C/min, where it was maintained for 5 min. The vaporizing chamber and FID detector were maintained at 240°C. The flow rates of the three carrier gases were 400 ml/min (air), 40 ml/min (hydrogen), and 20 ml/min (nitrogen). A standard curve was plotted based on the peak area ratio between the standard solutions of SCFAs (acetic acid, propionic acid, isobutyric acid, butyric acid, isovaleric acid, and valeric acid) and crotonic acid, and the content of SCFAs in each sample was calculated.

#### 2.3.7 16S rRNA sequencing

A DNA extraction kit was used to extract DNA from the colon contents of the mice. The V3-V4 hypervariable region of the 16S rRNA gene was amplified by PCR using the primers 341F (5’-CCTAYGGGRBGCASCAG-3′) and 806R (5’-GGACTACHVGGGTWTCTAAT-3′). Purified amplicons were paired-end sequenced on an Illumina NovaSeq PE250 platform. The raw sequencing reads were deposited into the NCBI Sequence Read Archive (SRA) database (Accession Number: PRJNA1040442).

### 2.4 Data and statistical analysis

SPSS 23 was used for statistical analysis, and GraphPad Prism 8.0.1 was used for mapping. Bacterial community analysis and mapping were carried out on the diversity cloud platform of Shanghai OE Biotech Co., Ltd. The Shapiro–Wilk test was used to assess the normality of data distribution. When comparing multiple groups, the Kruskal–Wallis test was performed to analyze data showing a non-normal distribution, and one-way ANOVA and Tukey’s *post hoc* comparison test were performed on data showing a normal distribution.

## 3 Results

### 3.1 Analysis of body weight and DAI

**Figure 2:**
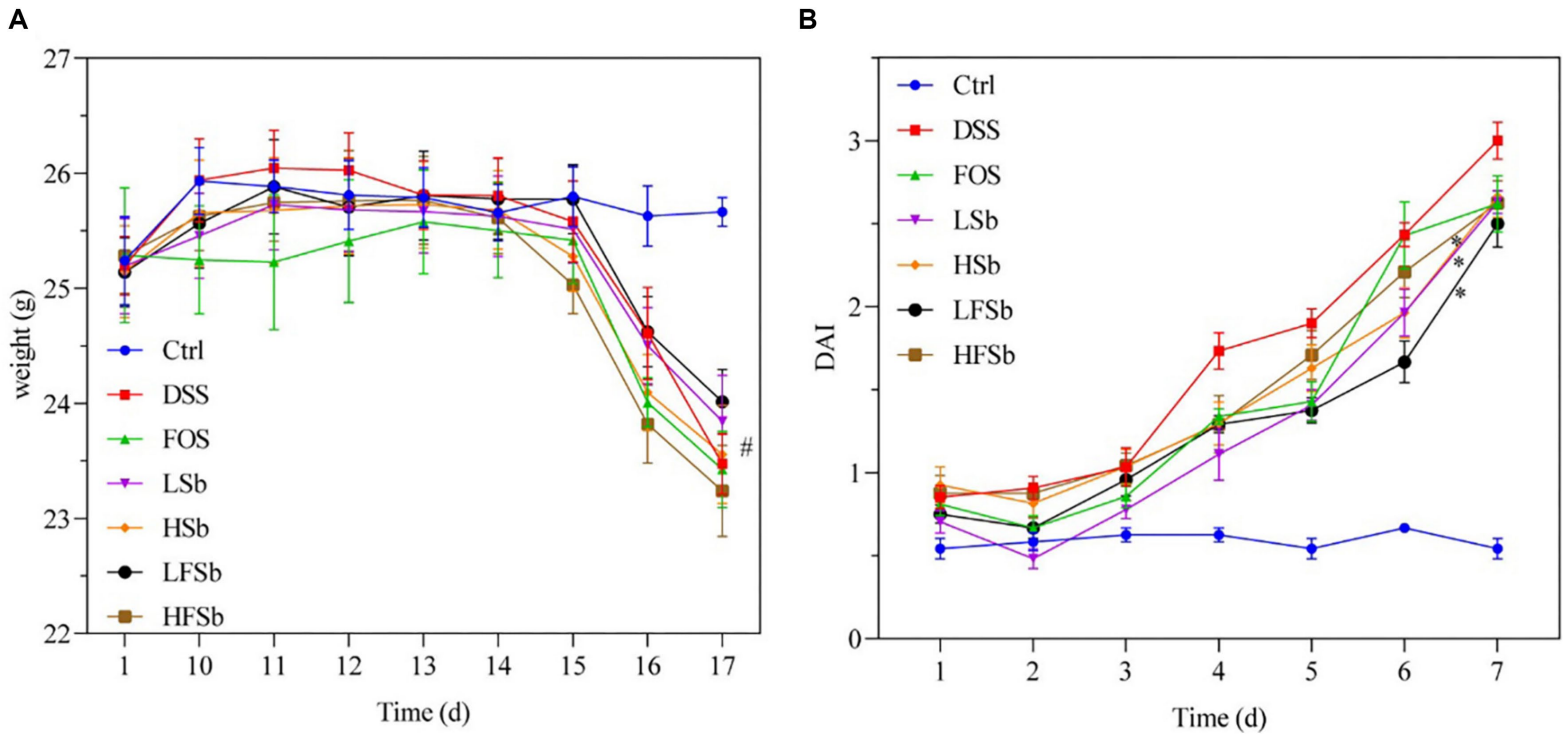
Effect of FOS and *Saccharomyces boulardii* on the body weight and disease activity index of the mice. **(A)** Body weight; **(B)** Disease activity index. Data are represented as the mean ± SEM. The Shapiro–Wilk test was used to evaluate whether the data were normally distributed. Subsequently, one-way ANOVA and Tukey’s *post hoc* comparison test were conducted. # indicates a significant difference compared with the Ctrl group: # indicates 0.01 < *p* < 0.05; * indicates a significant difference compared with the DSS group: * indicates 0.01 < *p* < 0.05.

The body weight changes in the mice during the experimental period are shown in Figure 2A. On day 10 (the end of T1), the average body weight of the mice increased in all other groups except the FOS group increased. On day 15 (day 6 after DSS intervention), the body weight of the mice in the other groups was significantly lower than that in the Ctrl group (*p* < 0.05), consistent with previous findings (Liu et al., 2023). On day 16 (day 7 after DSS intervention), all mice except those in the Ctrl group excreted loose and bloody stools. Their body weights decreased significantly, while their DAI values increased significantly. The DAI values of mice in all other groups were significantly higher than those in the Ctrl group on day 12 (day 3 after DSS intervention) (Figure 2B). On day 16 (day 7 after DSS intervention), only the DAI values in the LSb (*p* = 0.017), LFSb (*p* = 0.012), and HFSb groups (*p* = 0.036) were significantly lower than those in the DSS group.

### 3.2 Analysis of proinflammatory cytokine levels in the serum

**Figure 3:**
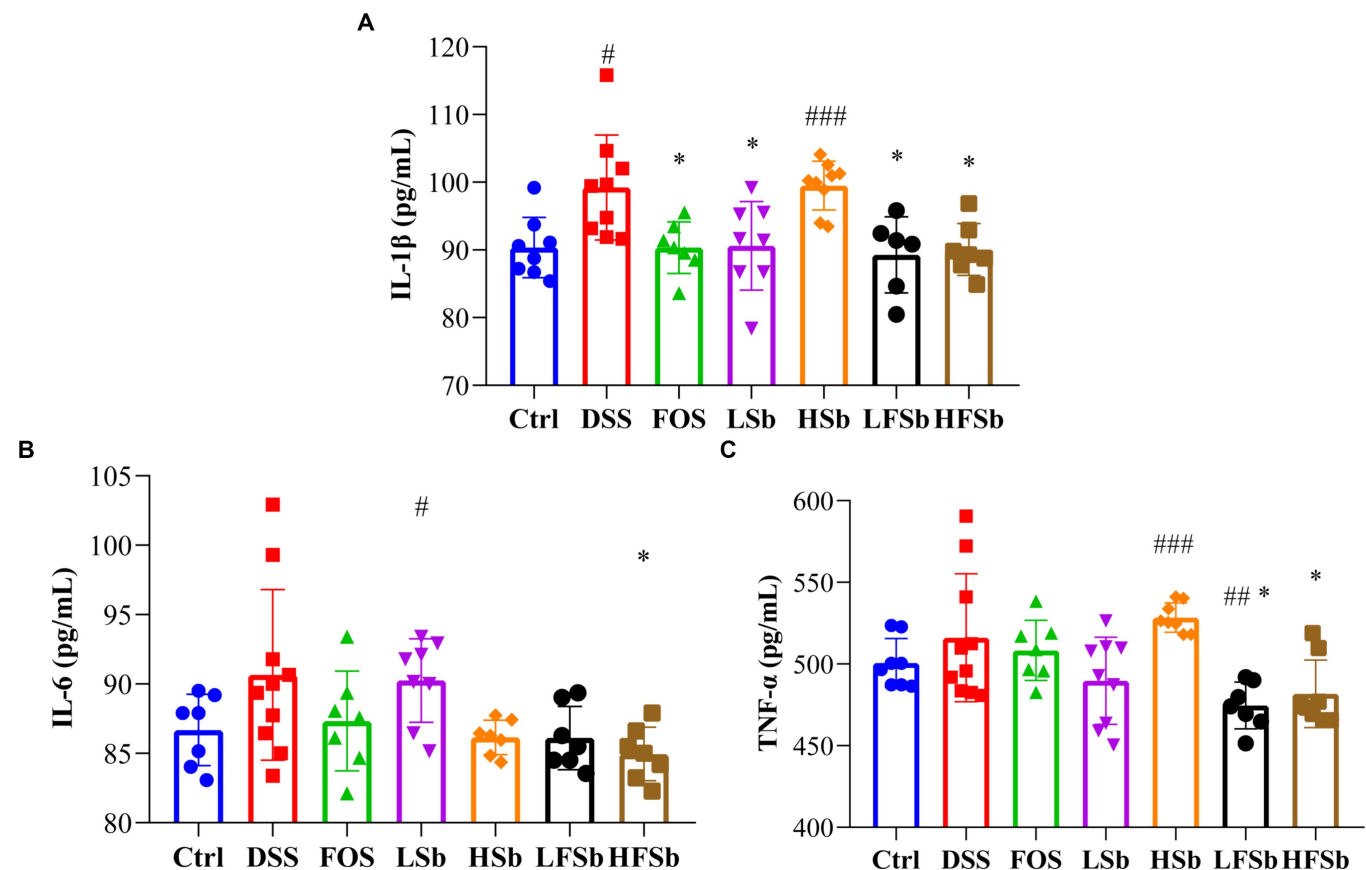
Serum levels of proinflammatory cytokines in the mice. **(A)** IL-1β; **(B)** IL-6; **(C)** TNF-α. Data are represented as the mean ± SD. The Shapiro–Wilk test was used to evaluate whether the data were normally distributed. Subsequently, one-way ANOVA and Tukey’s *post hoc* comparison tests were conducted. # Indicates a significant difference compared with the Ctrl group; # indicates 0.01 < *p* < 0.05, ## indicates 0.001 < *p* < 0.01, and ### indicates *p* < 0.001; * indicates a significant difference compared with the DSS group: * indicates 0.01 < *p* < 0.05.

The serum levels of proinflammatory factors were detected using ELISA in all groups of mice. The serum IL-1β concentration was significantly higher in mice from the DSS group (*p* = 0.012) and HSb groups (*p* < 0.001) than in those from the Ctrl group. Meanwhile, the serum IL-1β concentration was significantly lower in the FOS group (*p* = 0.015), LSb group (*p* = 0.026), LFSb group (*p* = 0.018), and HFSb group (*p* = 0.013) than in the DSS group (Figure 3A). Compared with the Ctrl group, the LSb group showed significantly elevated serum IL-6 levels (*p* = 0.029). Meanwhile, the serum IL-6 levels (*p* = 0.032) in the HFSb group were significantly lower than those in the DSS group (Figure 3B). Compared with the Ctrl group, the LFSb group showed significantly decreased serum TNF-α levels (*p* = 0.005), but the serum TNF-α levels were significantly increased in the HSb group (*p* < 0.001). The serum TNF-α levels in the LFSb group (*p* = 0.018) and the HFSb group (*p* = 0.040) were significantly lower than those in the DSS group (Figure 3C).

### 3.3 Colon characteristics and histology

**Figure 4:**
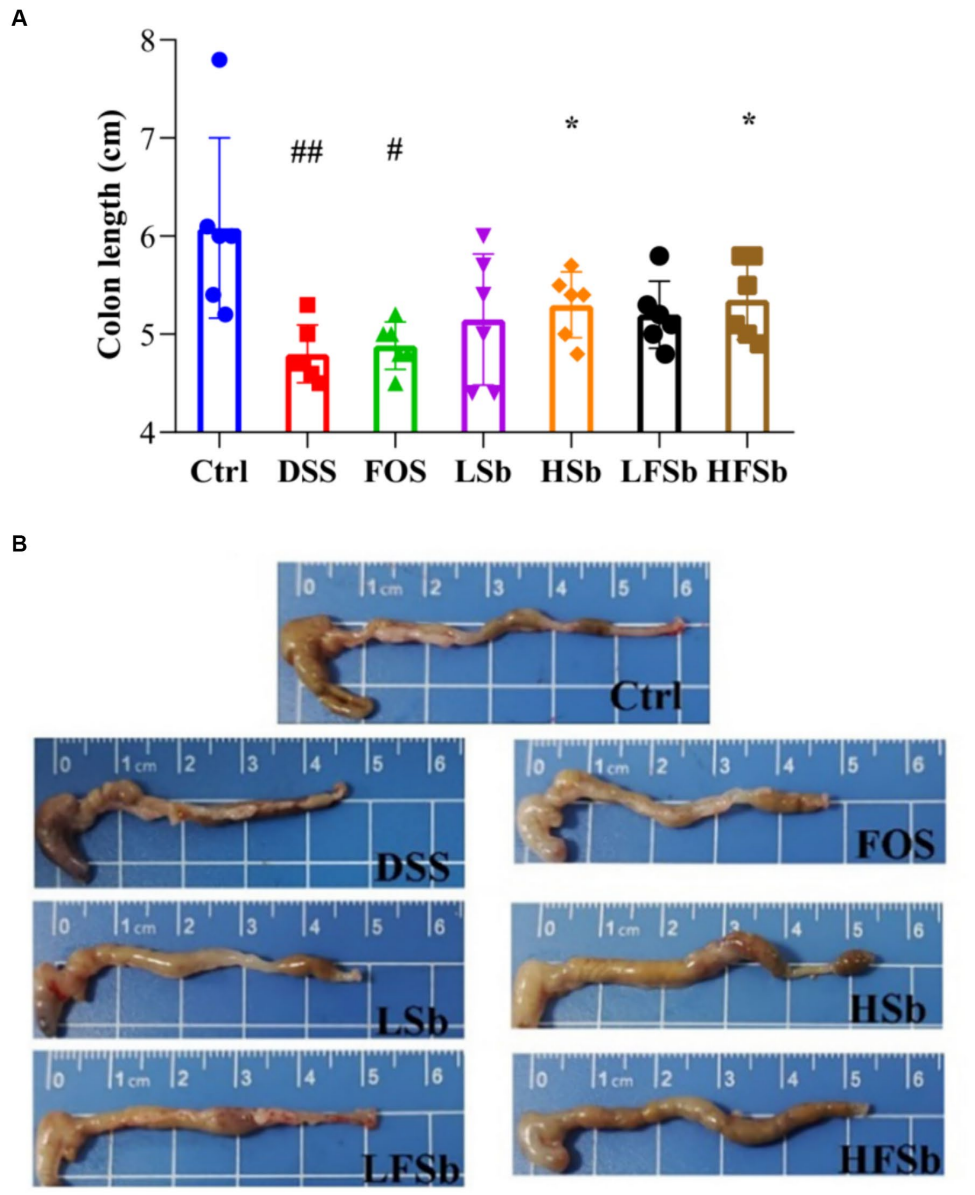
Colon length and colon morphology in mice from different groups. **(A)** Colon length; **(B)** Colon morphology. Data are represented as the mean ± SD. The Shapiro–Wilk test was used to evaluate whether the data were normally distributed. Subsequently, one-way ANOVA and Tukey’s *post hoc* comparison tests were conducted. # Indicates a significant difference compared with the Ctrl group: # Indicates 0.01 < *p* < 0.05, and ## indicates 0.001 < *p* < 0.01; * indicates a significant difference compared with the DSS group: * indicates 0.01 < *p* < 0.05.

An important manifestation of DSS-induced colitis in mice is a shortened colon. The more severe the inflammation, the shorter is the colon length (Fonseca-Camarillo and Yamamoto-Furusho, 2015). Hence, colon length and morphology were compared among all groups in this study (Figure 4). Compared with the Ctrl group, the other groups showed a shorter colon length; notably, the colon length of mice in the DSS group (*p* = 0.009) and the FOS group (*p* = 0.011) was significantly reduced (Figure 4). Compared with the DSS group, the HSb group (*p* = 0.021) and HFSb group (*p* = 0.023) showed a significantly higher colon length, although no significant difference in colon length was observed between the LSb and LFSb groups (Figure 4).

**Figure 5:**
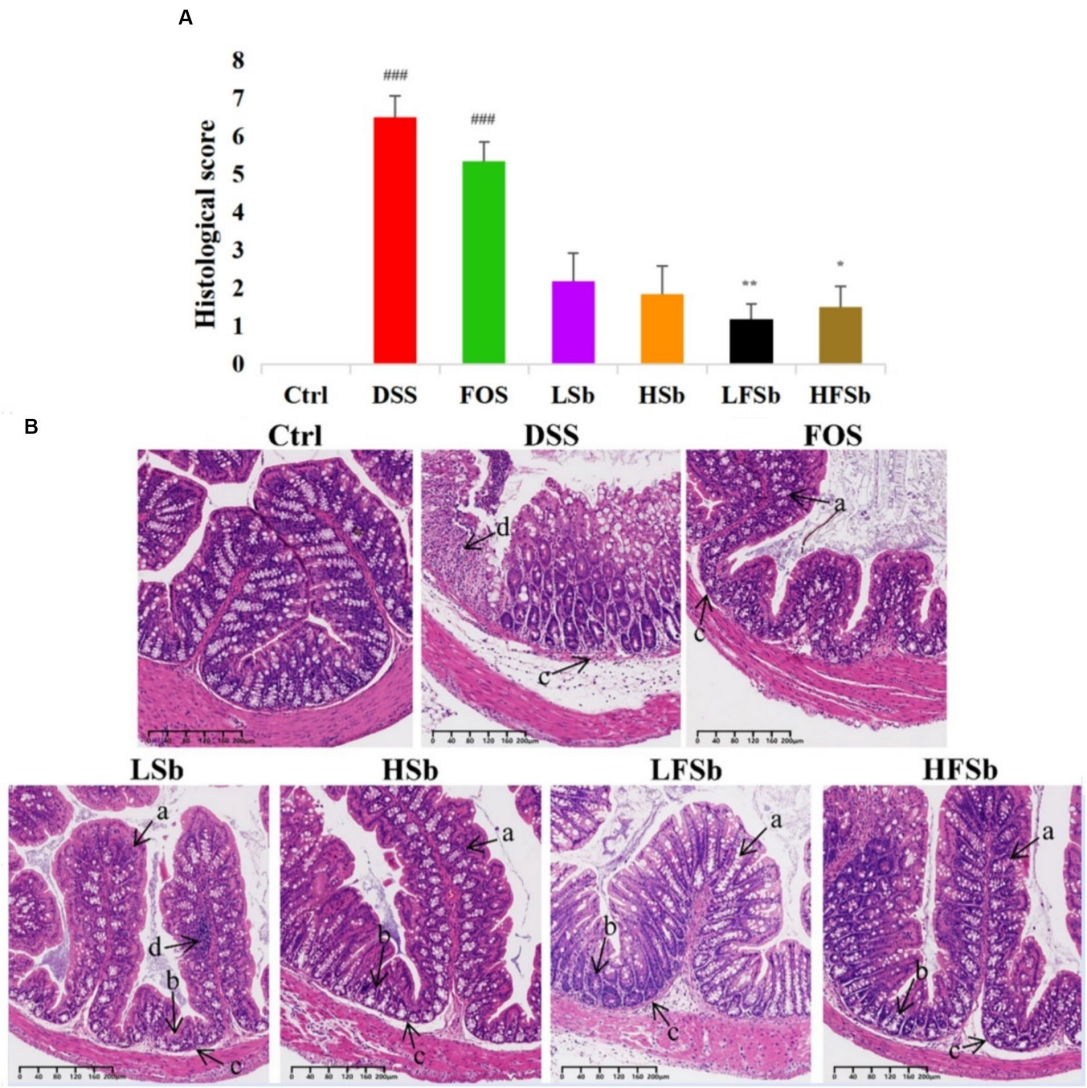
Histological analysis of the colon tissues of mice from different groups. **(A)** Histological scores; **(B)** Staining of colon tissues. Data are represented as the mean ± SEM. The Shapiro–Wilk test was used to evaluate whether the data were normally distributed. Subsequently, one-way ANOVA and Tukey’s *post hoc* comparison test were conducted. # Indicates a significant difference compared with the Ctrl group: ### means *p* < 0.001; * indicates a significant difference compared with the DSS group: * indicates *p* < 0.05, ** Indicates *p* < 0.01. (a) Goblet cells; (b) Crypts; (c) Submucosa; (d) Neutrophils.

By observing the microscopic morphology of colon tissues — such as intestinal villi, goblet cells, and crypts — the degree of inflammation can be estimated (Monk et al., 2018; Li M. X. et al., 2022). Therefore, colon tissues were histologically analyzed using H&E staining according to the modified Riley Scoring System (Figure 5). Healthy mice in the Ctrl group showed normal colon tissue morphology. However, DSS-induced colitis damaged the integrity of the colon mucosa. In animals with DSS-induced colitis, the goblet cells either decreased in number or completely disappeared. In these animals, the crypt was deformed, atrophied, or even absent. There were ulcers in the submucosa and neutrophil infiltration was also detected. These results indicated the successful establishment of the mouse colitis model.

The colonic morphology of mice in the LFSb and HFSb groups showed the most significant improvement when compared with the DSS group. In these groups (*p* < 0.01, *p* < 0.05), goblet cells with mucus-secreting functions were largely preserved. Meanwhile, the structural integrity of the crypts and submucosa was also significantly improved, and inflammatory cell infiltration was only detected locally in the mucosa.

The colon morphology of mice in the LSb and HSb groups was also improved to a certain extent, with intestinal villi and cup cells being partially retained. However, in these groups, some crypts were deformed and atrophied, and considerable local inflammatory cell infiltration was detected. The histological scores in the LSb and HSb groups were not significantly better than those in the DSS group. In contrast, the villi in the FOS group were significantly atrophied. In this group, the goblet cells had largely disappeared, and the crypts were also deformed or had disappeared. With the exception of the DSS group, the FOS group had the highest histological score. Furthermore, the histological scores of the DSS and FOS groups were both significantly higher than those of the control group (*p* < 0.001). In summary, a significant amelioration of colon injury was observed in the LFSb and HFSb groups. While the LSb and HSb groups also showed some degree of improvement, the improvement was not as high as that in the LFSb and HFSb groups.

### 3.4 Analysis of SCFA content in feces

**Figure 6:**
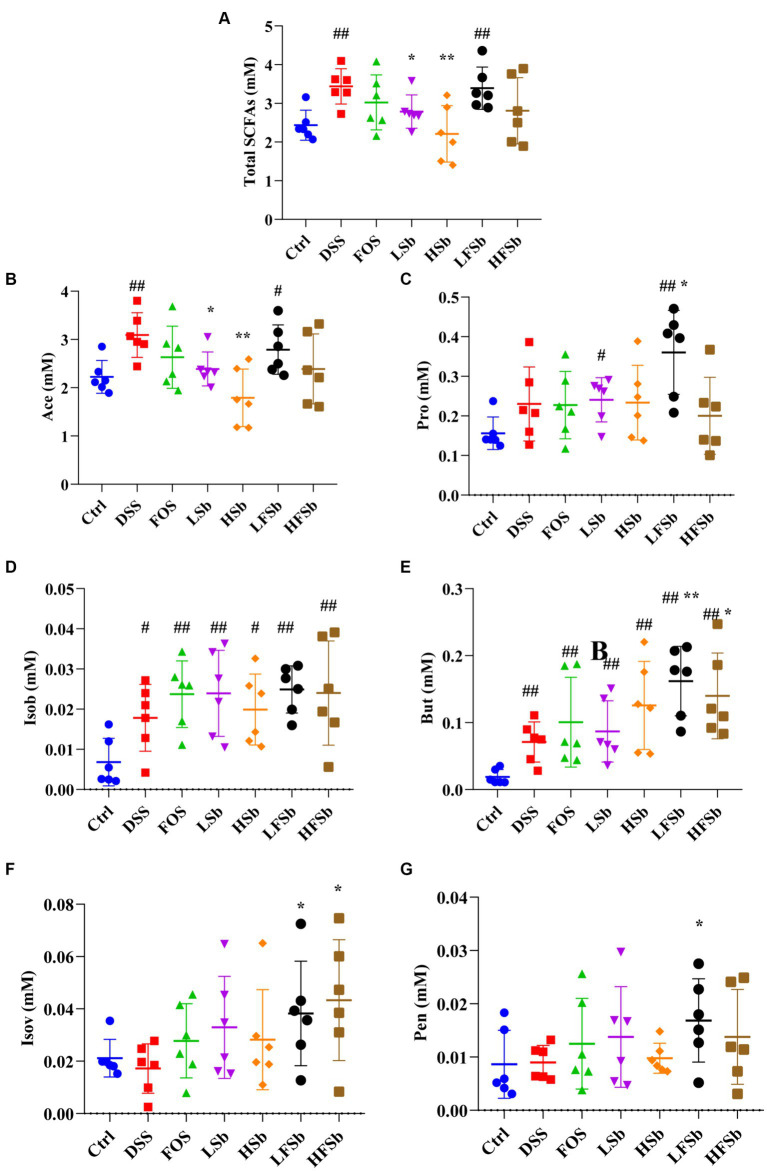
Effects of FOS and *S. boulardii* on short-chain fatty acid (SFCA) contents in the feces of different groups. **(A)** Total SCFA content, i.e., total contents of six SCFAs; **(B)** Ace (acetic acid); **(C)** Pro (propionic acid); **(D)** Isob (isobutyric acid); **(E)** But (butyric acid); **(F)** IsoV (Isovaleric acid); **(G)** Pen (valeric acid). Data are represented as the mean ± SD. The Shapiro–Wilk test was used to evaluate whether the data were normally distributed. Subsequently, one-way ANOVA and Tukey’s *post hoc* comparison tests were conducted. # Indicates a significant difference compared with the Ctrl group: # indicates 0.01 < *p* < 0.05, and ## indicates 0.001 < p < 0.01; * indicates a significant difference compared with the DSS group: * indicates 0.01 < *p* < 0.05, and ** indicates 0.001 < *p* < 0.01.

The excrements of mice were collected, and six types of SCFAs were detected in mouse stool samples. Standard solutions for six concentration gradients of the SCFAs (acetic acid, propionic acid, isobutyric acid, butyric acid, isovaleric acid, and valeric acid) were prepared. Subsequently, standard curves were generated based on the peak area ratio and concentration ratio between each SCFA and crotonic acid. Fecal samples were subjected to various hydrolysis and methyl esterification conditions (different durations, temperatures, and gas usage conditions) to obtain maximum extraction efficiency. The total SCFA content was significantly higher in the DSS (*p* = 0.002) and the LFSb group (*p* = 0.006) than in the Ctrl group. However, it was significantly lower in the LSb (*p* = 0.030) and the HSb group (*p* = 0.006) than in the DSS group (Figure 6A). The proportion of acetic acid in the fecal samples was much higher than that of the other SCFAs. The between-group differences in the levels of these individual SCFAs were similar to the between-group differences in the total SCFA content (Figure 6B). The propionic acid content in the LSb (*p* = 0.015) and LFSb groups (*p* = 0.004) was significantly higher than that in the Ctrl group. Moreover, the propionic acid levels in the LFSb group (*p* = 0.047) were significantly higher than those in the DSS group (Figure 6C). The contents of butyric acid and isobutyric acid in the DSS group and the other five experimental groups were significantly higher than those in the Ctrl group, while the contents of butyric acid in the LFSb (*p* = 0.004) and HFSb groups (*p* = 0.038) were significantly higher than those in the DSS group (Figures 6D,E). In addition, the contents of valeric acid (*p* = 0.045) and isovaleric acid (*p* = 0.042) in the LFSb group and the contents of isovaleric acid (*p* = 0.028) in the HFSb group were significantly higher than those in the DSS group (Figures 6F,G).

### 3.5 Analysis of gut microbial composition

**Figure 7:**
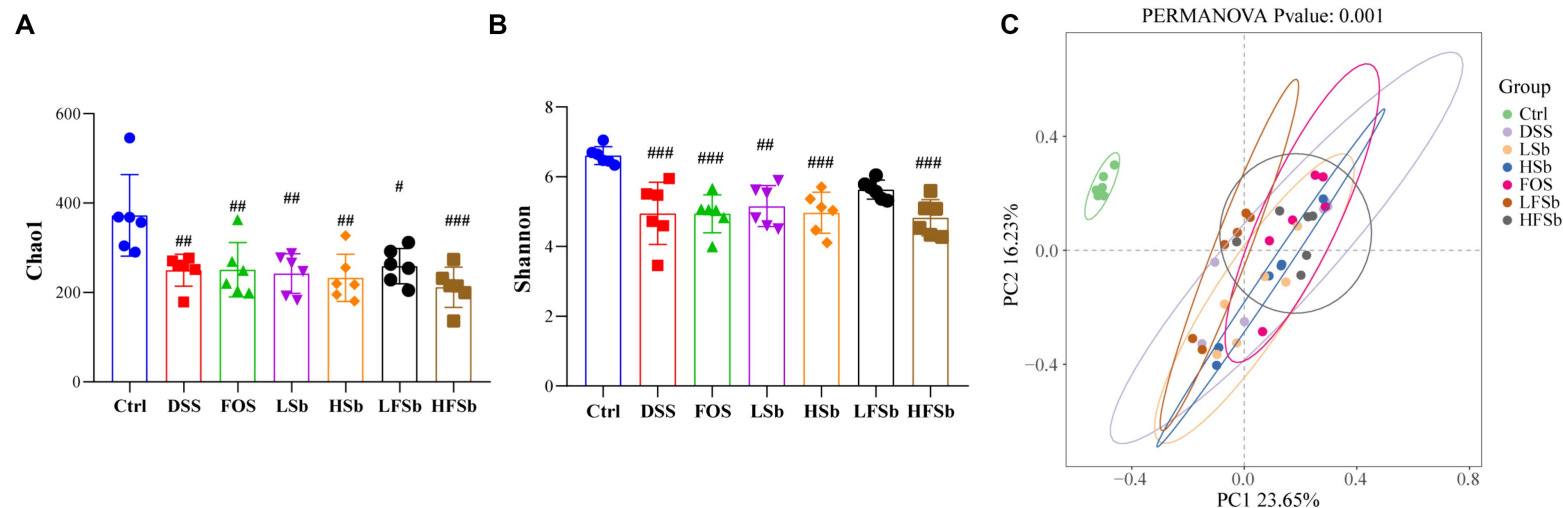
Microbial composition analysis of primary fecal samples and fermentation samples at the genus level. **(A)** Alpha-diversity analysis based on the Chao1 index; **(B)** Alpha-diversity analysis based on the Shannon index; **(C)** Beta-diversity analysis based on Bray-Curtis distances and PCoA; Data are represented as the mean ± SD. The Shapiro–Wilk test was used to evaluate whether the data were normally distributed. Subsequently, one-way ANOVA and Tukey’s *post hoc* comparison tests were conducted. # Indicates a significant difference compared with the Ctrl group: # indicates 0.01 < *p* < 0.05, ## indicates 0.001 < *p* < 0.01, and ### indicates *p* < 0.001.

The colon contents of mice were sequenced using 16S rRNA gene sequencing, and the composition of the gut microbiota in different groups of mice was analyzed. First. the α-diversity of the colon microbiota was examined (Figures 7A,B). Compared with Ctrl treatment, the DSS intervention significantly reduced the Chao1 index and Shannon index of the gut microbiota in mice. Thus, DSS-induced colitis significantly reduced the abundance and diversity of the gut microbiota. In addition, a significant decrease in the Shannon index was observed in all other treatment groups except the LFSb group. This indicated that LFSb could partly attenuate the decrease in gut microbiota diversity. However, there was no significant difference in the abundance and diversity of the gut microbiota between the other five experimental groups and the DSS group.

Principal coordinate analysis (PCoA) revealed a distinct separation between the ꞵ-diversity of the Ctrl group and that of the other six groups. This indicated that after the DSS intervention, a significant alteration in the gut microbiota was in all groups (when compared with the Ctrl group) (Figure 7C). However, there was a small difference between the DSS group and the other five experimental groups. In addition, there was no overlap between the FOS group and the LFSb group, indicating a significant variation in the composition of the gut microbiota between these two groups (Figure 7C).

**Figure 8:**
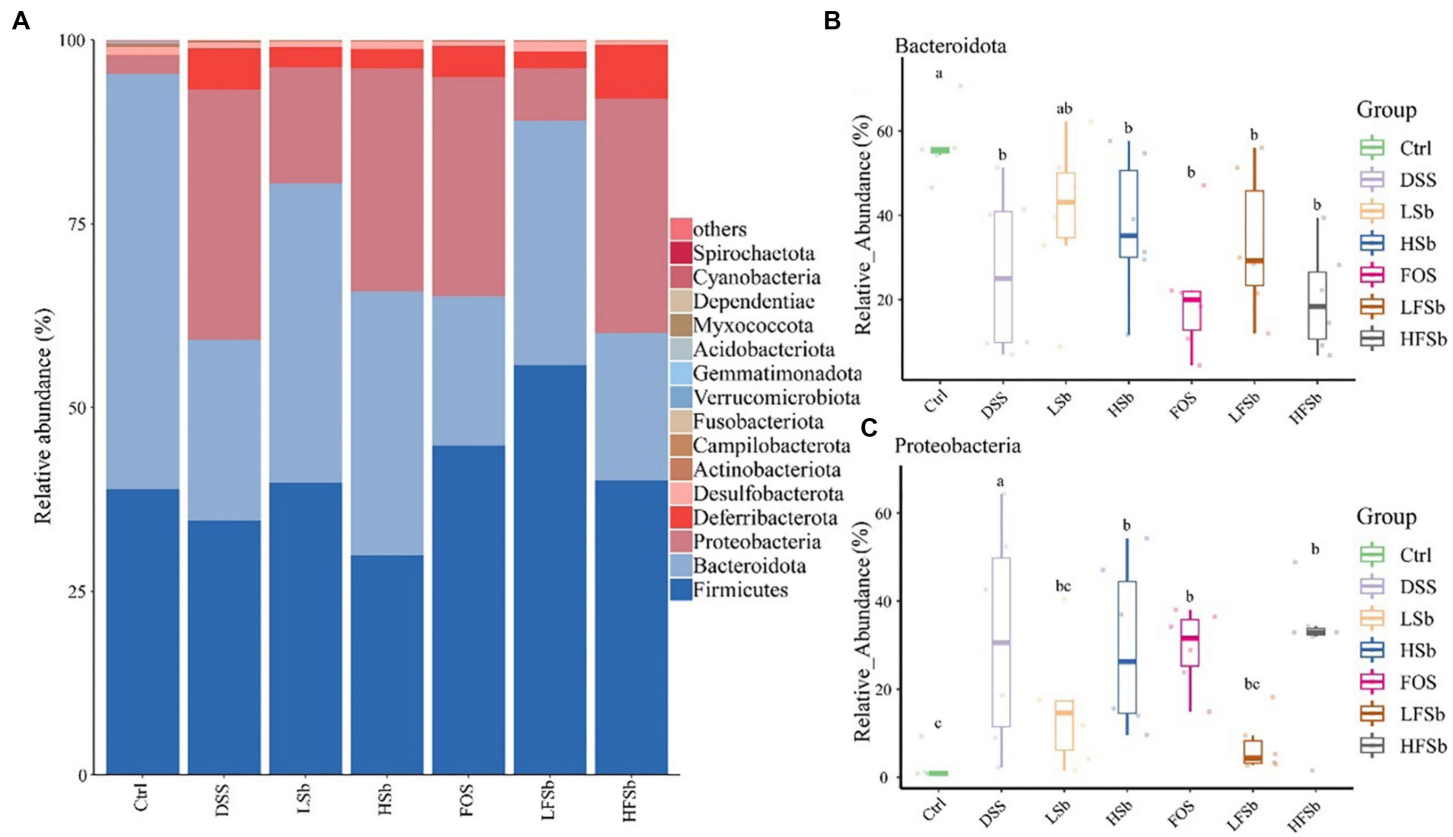
Colon microbiota composition and group differences at the phylum level. **(A)** Microbial composition at the phylum level; Differences in the relative abundance of Bacteroidota **(B)** and Proteobacteria **(C)** between different groups. Data are represented as the mean ± SD. The Shapiro–Wilk test was used to evaluate whether the data were normally distributed. Subsequently, the Kruskal-Wallis test was conducted. a, b, c: different lowercase letters represent significant differences between different groups.

The abundance of intestinal bacteria was examined at the phylum level. Firmicutes and Bacteroidota were the dominant phyla in the colons of healthy mice, accounting for more than 95% of the total microbes, followed by Actinobacteriota and Desulfobacterota (Figure 8A). The relative abundance of Deferribacterota increased significantly after DSS intervention, and this was one of the dominant phyla in the DSS group.

The mean relative abundance of Firmicutes in the LFSb group was higher than that in the other groups, but there was no significant difference in its abundance among the other groups. Notably, there were significant differences in the relative abundance of Bacteroidota and Proteobacteria among the groups. The abundance of Bacteroidota in all other groups except the LSb group was significantly lower than that in the Ctrl group (Figure 8B). Meanwhile, the relative abundance of Proteobacteria in the DSS group, FOS group, HSb group, and HFSb group was significantly higher than that in the Ctrl group. In contrast, the relative abundance of Proteobacteria in these five experimental groups was significantly lower than that in the DSS group (Figure 8C).

**Figure 9:**
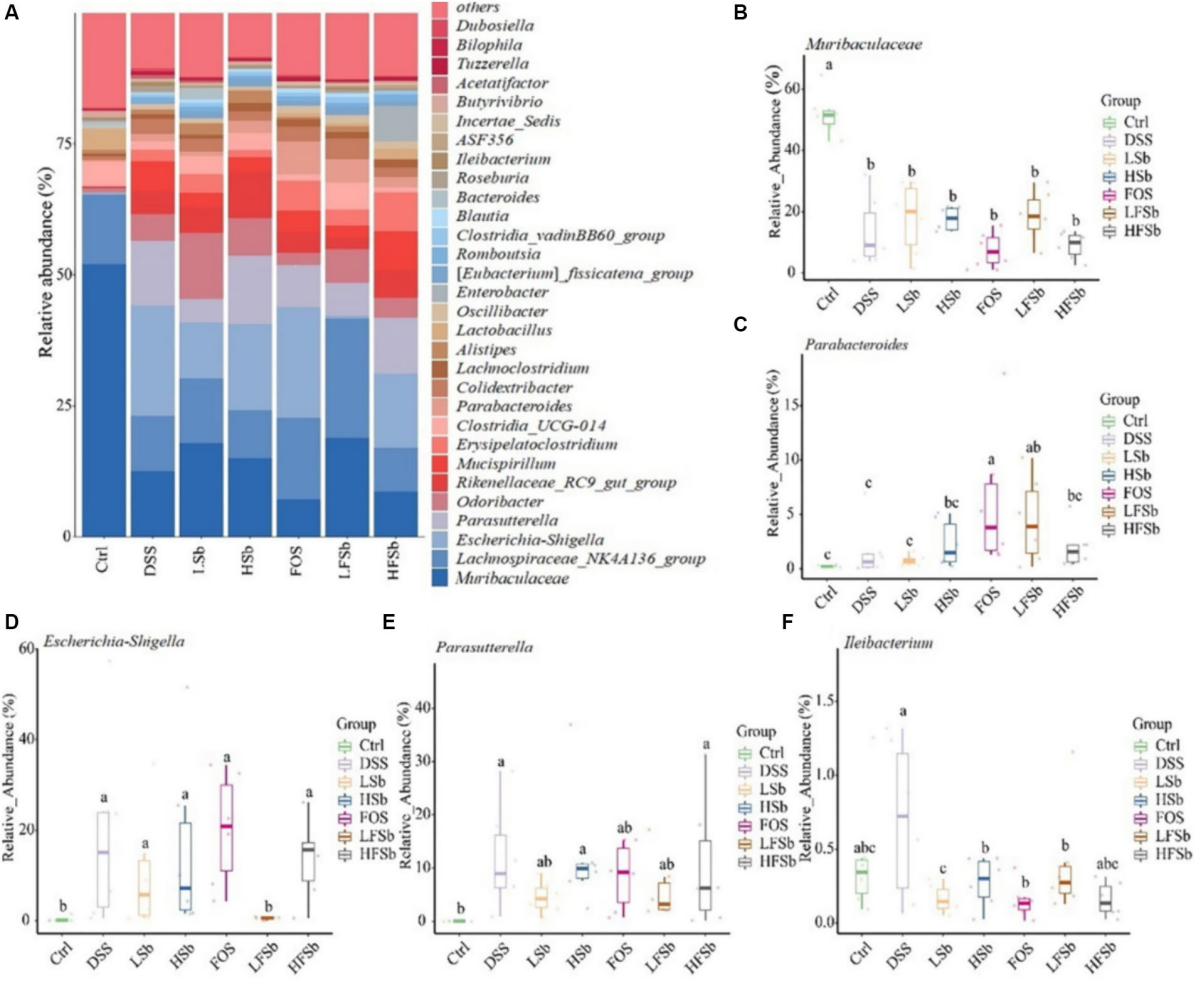
Colon microbiota composition and group differences at the genus level. **(A)** Microbial composition at the genus level; **(B–F)** Differences in *Muribaculaceae*, *Parabacteroides*, *Escherichia−Shigella*, *Parasutterella*, and *Ileibacterium* between groups. Data are represented as the mean ± SD. The Shapiro–Wilk test was used to evaluate whether the data were normally distributed. Subsequently, the Kruskal–Wallis test was conducted. a, b, c: different lowercase letters represent significant differences between different groups.

The top 30 genera (based on relative abundance) in the mouse gut are shown in Figure 9A. *Muribaculaceae* was the genus with the highest relative abundance in the colons of healthy mice, accounting for more than 50% of the total microbes. It was followed by *Lachnospiraceae_NK4A136_group* (13.35%), *Clostridia_UCG-014* (4.70%), and *Lactobacillus* (3.83%). The composition of the microbiota in the other groups was significantly different from that in the Ctrl group, with *Muribaculaceae* of Bacteroidota showing the most significant change (Figure 9B). The relative abundance of *Parabacteroides* in the FOS and LFSb groups was significantly higher than that in the Ctrl and DSS groups (Figure 9C). The relative abundance of *Escherichia-Shigella* was significantly elevated in all other groups except the LFSb group (Figure 9D). Meanwhile, the relative abundance of *Parasutterella* increased significantly in the DSS, HSb, and HFSb groups, when compared with the Ctrl group, and also tended to increase in the FOS, LSb, and LFSb groups (Figure 9E). The relative abundance of *Ileibacterium* in the FOS, LSb, HSb, and LFSb groups was significantly lower than that in the DSS group (Figure 9F).

## 4 Discussion

The present study found that typically, FOS and *S. boulardii* alone cannot prevent the occurrence of DSS-induced colitis. However, FOS/*S. boulardii* mixed suspensions (10^7^ CFU/ml and 10^9^ CFU/ml) and the 10^7^ CFU/ml *S. boulardii* suspension may significantly attenuate the increased DAI in mice after DSS treatment and alleviate the clinical symptoms of colitis to a certain extent.

A previous study reported that 10^9^ CFU/ml of *S. boulardii* may also reduce the DAI and prevent colon damage in DSS-induced mouse models of colitis. However, this study did not compare the effects of different concentrations of *S. boulardii* (Rodriguez-Nogales et al., 2018). Gao H et al. found that *S. boulardii* suspensions of 10^5^ CFU/mL may significantly prevent weight loss in DSS-induced mouse models of colitis, improve the DAI, and reduce the colon weight/length ratio (Gao H. et al., 2021). Meanwhile, different concentrations of *S. boulardii* have also been shown to reduce colon inflammation to varying extents, and their combination with FOS has been found to enhance this improvement.

IL-1β, IL-6, and TNF-α are important cytokines that regulate inflammation and their release triggers and promotes the inflammatory response. Therefore, these cytokines are often used as inflammatory markers to estimate the severity of inflammation in the body (Qu S. et al., 2021; Nakase et al., 2022). In the present study, the HFSb group showed significantly reduced levels of these proinflammatory cytokines. The anti-inflammatory effect was the strongest in the HFSb group, (significant reductions in IL-6, IL-1β, and TNF-α), followed by the LFSb group (significant reductions in IL-1β and TNF-α). Meanwhile, the FOS and LSb groups only exhibited significantly reduced levels of IL-1β, and the HSb group did not show any obvious alteration in proinflammatory cytokines. Gao et al. reported that *S. boulardii* suspensions of both 10^5^ CFU/mL and 10^7^ CFU/ml can reduce the levels of IL-1β, IL-6, and TNF-α. This indicates that a high concentration of *S. boulardii*, such as 10^9^ CFU/ml, may indeed have a poor effect on improving inflammation (Gao H. et al., 2021). Zhang et al. discovered that FOS combined with GOS can decrease the serum levels of IL-1β, IL-6, IL-8, and TNF-α in mice with DSS-induced colitis (Zhang S. et al., 2023). Meanwhile, Rajkumar et al. found that the combination of *Lactobacillus salivarius* and FOS can significantly lower the serum levels of IL-1β, IL-6, IL-8, and TNF-α in adults, and combination treatment provides more obvious treatment effects than *Lactobacillus salivarius* alone (Rajkumar et al., 2015). Therefore, the combination of FOS and *S. boulardii* appears to be more effective at inhibiting the release of proinflammatory cytokines than either of these agents alone. Moreover, the concentration of *S. boulardii* also seems to affect the inhibitory effect of this probiotic on proinflammatory cytokines.

The SCFAs present in mouse feces represent the residual products that are synthesized by gut microbes but not absorbed by the body. Notably, the integrity of the intestinal mucosa determines the absorption efficiency of SCFAs (Zhou et al., 2020; Guo et al., 2021). DSS-induced colitis can cause intestinal mucosal damage in mice, thus inhibiting nutrient absorption. This may partly explain the increase in SCFA content in the feces of mice from the DSS group (Niu et al., 2023; Wu et al., 2023). SCFAs, especially butyric acid, have a strong anti-inflammatory effect. Hence, they can help maintain the integrity of the intestinal mucosal barrier and thus improve colon health (Parada Venegas et al., 2019; Li W. et al., 2022). In this study, the content of butyric acid was significantly higher in the LFSb and HFSb groups than in the DSS group, which further explains why the intestinal damage was the least severe in these two groups. In addition to butyric acid, a variety of other SCFAs also showed significantly higher levels in fecal samples from the LFSb group than in those from the Ctrl group. The combination of FOS and *S. boulardii* promoted the production of SCFAs by intestinal microbes and the SCFA content in the LFSb group was higher than that in the LSb and FOS groups. These results indicated that FOS combined with *S. boulardii* was more effective at promoting fermentation than either agent alone. This result was consistent with the conclusion derived by Morales-Ferre C et al. regarding the preventive effects of probiotics and prebiotics on early rotavirus-induced diarrhea in rats. Combination of probiotics and prebiotics, such as FOS and *S. boulardii*, can significantly reduce intestinal permeability and increase the levels of SCFAs when compared with single-agent treatment (Morales-Ferre et al., 2022). However, in the present study, some differences were detected between the LSb group and HSb group and between the LFSb group and HFSb group, indicating that the concentration of *S. boulardii* also plays a key role in regulating fermentation activity in gut microbiota.

By examining the gut microbiota composition in mice, this study showed that DSS could inhibit Bacteroidota and promote the growth of Proteobacteria. Meanwhile, LSb could attenuate these changes and maintain the balance of the gut microbiota. Notably, LFSb could also inhibit the growth of Proteobacteria and promote the growth of Firmicutes to a certain extent. These findings were consistent with the results obtained by Yu L et al. in their intervention study that focused on liver injury induced by D-Galactosamine in mice. These researchers found that *S. boulardii* significantly increases the relative abundance of Bacteroidota, and decreases the relative abundance of Firmicutes and Proteobacteria (Yu et al., 2017).

Compared with the Ctrl group, the other groups also showed a significantly reduced relative abundance of *Muribaculaceae* from the phylum Bacteroidota. Interestingly, *Muribaculaceae* is known to have a probiotic effect and can influence mucus layer formation and barrier function in the colon (Mu et al., 2021; Zhang Y. et al., 2023). In the present study, its mean relative abundance was the lowest in the DSS group and the FOS group, which may be one reason why colitis injury was more severe in these two groups. Furthermore, *Parabacteroides* from the phylum Bacteroidota has been shown to inhibit the inflammatory response by regulating IL-10 levels and Treg cells (Strandwitz et al., 2019; Koh et al., 2020). The relative abundance of *Parabacteroides* in the FOS and LFSb groups was significantly higher than that in the Ctrl and DSS groups, which may partly explain why inflammation was alleviated in the LFSb group. Although the abundance of *Parabacteroides* was also significantly increased in the FOS group, the other microbial indicators in this group were poor. Hence, the findings suggested that the anti-inflammatory effect in the FOS group was limited. *Muribaculaceae* and *Parabacteroides* have previously been identified as potential SCFAs-producing bacteria (Cui et al., 2022; Yang et al., 2023). Studies have demonstrated a strong positive correlation between *Muribaculaceae* and propionic acid production as well as a moderate association between *Muribaculaceae* and butyric acid production (Pei et al., 2023). Meanwhile, *Parabacteroides* has been shown to have a positive correlation with the level of acetic acid production (He et al., 2023).

*Escherichia–Shigella* from the phylum Proteobacteria can increase intestinal permeability and exacerbate colitis (Zorraquin-Pena et al., 2021). *Escherichia–Shigella are* negatively correlated with SCFA production (Huang et al., 2023). Among all the treatment groups in this study, only the LFSb group showed no significant increase in the abundance of *Escherichia–Shigella*. This suggested that LFSb effectively inhibited the growth of the harmful bacteria *Escherichia–Shigella*, thereby protecting the intestinal tract and reducing damage due to colitis. The relative abundance of *Parabacteroides* and *Escherichia–Shigella* was similar between the HFSb and DSS groups. However, HFSb did not provide the same effects as LFSb, which indicated that the concentration of *S. boulardii* affects the efficacy of microbiota regulation. Previously, the increase in *Parasutterella* (phylum Proteobacteria) in the gut has been linked to dysbiosis and decreased microbial diversity (Jang et al., 2019). In this study, although the FOS, LSb, and LFSb groups showed a higher relative abundance of *Parasutterella* than the Ctrl group, this difference was not significant. This may be one reason why the microbial diversity in the LFSb group did not decrease remarkably. Furthermore, the relative abundance of *Ileibacterium* in the FOS, LSb, HSb, and LFSb groups was significantly lower than that in the DSS group. *Ileibacterium* is a genus from the phylum Firmicutes, and it is a harmful group of bacteria. *Ileibacterium* has previously been linked to metabolic disorders and intestinal inflammation (Gao X. et al., 2021; Lin et al., 2022). However, some studies have shown that *Ileibacterium* and *Parasutterella* are positively correlated with butyric acid production (Huang et al., 2023; Cao et al., 2024). In the present study, the abundance of *Ileibacterium* and *Parasutterella* in the Ctrl group was significantly lower than that in the DSS group. This could explain why the butyric acid content in the Ctrl group was lower than that in the DSS group.

Although both the LFSb and HFSb groups showed a lower relative abundance of Proteobacteria than the DSS group, their levels were much lower in the LFSb group. In fact, the relative abundance of Proteobacteria was comparable between the LFSb group and the Ctrl group. Notably, only the relative abundance of *Escherichia–Shigella* and *Parasutterella* (both belonging to phylum Proteobacteria) was significantly lower in the LFSb group than in the DSS group, and there was no significant difference in the relative abundance of these bacteria between the LFSb group and the Ctrl group. The relative abundance of *Ileibacterium* (phylum Proteobacteria) in both groups was significantly lower than that in the DSS group, but only the LFSb group showed similar levels of *Ileibacterium* as the Ctrl group. In addition, compared with the DSS group, the relative abundance of *Parabacteroides* (phylum Bacteroidetes) was significantly elevated in the LFSb group, but not in the HFSb group. Overall, these results demonstrated that the concentration of *S. boulardii* has an important effect on modulating the reduction in intestinal inflammation and improving the gut microbiota, as suggested by several previous studies. Therefore, additional studies involving more concentration gradients of *S. boulardii* in combination with FOS are warranted. Overall, accurate evidence and theoretical confirmation are required for the combined use of these agents in clinical settings.

## 5 Conclusion

Intervention with DSS can cause a gut microbiota imbalance and reduce the richness and diversity of the gut microbes. By establishing a DSS-induced colitis mouse model, this study analyzed the correlation between the composition and characteristics of intestinal microbiota and the levels of proinflammatory cytokines in the serum and SCFAs in feces, while accounting for the severity of colitis. Therefore, this study provided a potential strategy for managing colitis by regulating the intestinal microbiota. This study showed that 10^7^ CFU/ml of *S. boulardii* combined with FOS can reduce colitis-induced injury and protect the intestinal mucosal barrier by attenuating intestinal dysbiosis, promoting the growth of the beneficial bacteria *Parabacteroides*, and inhibiting the growth of the harmful bacteria *Escherichia–Shigella*. The effect of the combination was found to be obviously better than that of FOS or *S. boulardii* alone, and the concentration of *S. boulardii* appeared to be a key factor affecting the efficacy of the intervention. These results provide a scientific basis for the improved prevention and treatment of colitis using a FOS/*S. boulardii* combination. They also offer a theoretical basis for the development of preparations containing FOS combined with *S. boulardii*.

## Data availability statement

The datasets presented in this study can be found in online repositories. The names of the repository/repositories and accession number(s) can be found at: https://www.ncbi.nlm.nih.gov/, PRJNA1040442.

## Ethics statement

The animal study was approved by Institutional Animal Care and Use Committee (ACUC) of Zhejiang Academy of Agricultural Sciences (2022ZAASLA57). The study was conducted in accordance with the local legislation and institutional requirements.

## Author contributions

YW: Data curation, Methodology, Writing – original draft, Writing – review & editing. HF: Data curation, Formal analysis, Methodology, Writing – review & editing. XX: Data curation, Investigation, Validation, Writing – review & editing. HJ: Investigation, Methodology, Project administration, Resources, Writing – review & editing. Q-jK: Investigation, Methodology, Project administration, Resources, Writing – review & editing. W-lT: Investigation, Methodology, Project administration, Resources, Writing – review & editing. BW: Project administration, Supervision, Writing – review & editing. GZ: Funding acquisition, Supervision, Writing – review & editing. X-eP: Methodology, Project administration, Supervision, Writing – review & editing.
